# Interactions between circadian clocks and photosynthesis for the temporal and spatial coordination of metabolism

**DOI:** 10.3389/fpls.2015.00245

**Published:** 2015-04-09

**Authors:** Antony N. Dodd, Fiona E. Belbin, Alexander Frank, Alex A. R. Webb

**Affiliations:** ^1^School of Biological Sciences, University of Bristol, Bristol, UK; ^2^Cabot Institute, University of Bristol, Bristol, UK; ^3^Department of Plant Sciences, University of Cambridge, Cambridge, UK

**Keywords:** circadian rhythms, sugar signaling, photosynthesis, chloroplasts

## Abstract

All plant productivity, including the food that we eat, arises from the capture of solar energy by plants. At most latitudes sunlight is available for only part of the 24 h day due to the rotation of the planet. This rhythmic and predictable alteration in the environment has driven the evolution of the circadian clock, which has an extremely pervasive influence upon plant molecular biology, physiology and phenology. A number of recent studies have demonstrated that the circadian clock is integrated very closely with photosynthesis and its metabolic products. We consider the coupling of the circadian oscillator with carbohydrate biochemistry and the connections between the nuclear-encoded circadian clock and processes within chloroplasts. We describe how this might provide adaptations to optimize plant performance in an environment that varies both predictably upon a daily and seasonal basis, and unpredictably due to the weather.

## Introduction

Circadian rhythms are thought to allow plants to adapt to the cycles of daylight caused by the rotation of the planet. In the model plant *Arabidopsis thaliana*, biomass accumulation, photosynthesis, seed number and seed viability are increased by correct circadian regulation (Green et al., 2002; Dodd et al., 2005). Circadian regulation also contributes to agronomic traits of crops including cereals, rice and soybean (Turner et al., 2005; Izawa et al., 2011; Preuss et al., 2012). Recent studies have found that circadian regulation is integrated closely with photosynthesis and its metabolic products. We discuss recent advances in understanding of the processes that integrate circadian regulation with photosynthesis. We place this within the context of mechanisms that communicate circadian timing cues between subcellular compartments, between cells and tissues, and between organs.

## Circadian Rhythms

Circadian rhythms are daily rhythms of biological activity with a period of about 24 h that are able to persist in the absence of external cues and so allow anticipation of regular environmental alterations, such as the dawn/dusk cycle (Dodd et al., 2014). In addition to anticipation of light and dark transitions, the circadian clock synchronizes, sequences or temporally separates biologically-associated or incompatible processes (Harmer et al., 2000) and adapts plants to the progression of seasons (Hicks et al., 1996; Park et al., 1999) through modulation of gene expression and protein activity.

Higher plant circadian rhythms arise from a gene network termed the circadian oscillator. This incorporates autoregulatory and interlocked transcription-translation feedback loops. The nature and interconnectivity of the network produces under constant environmental conditions 24 h cycles of transcription of genes encoding oscillator components (Nagel and Kay, 2012). Light and temperature adjust the phase of the oscillator to track dawn and dusk through the process of entrainment (Dodd et al., 2014; Hsu and Harmer, 2014), which also matches oscillator phase to seasonal changes in the time of dawn.

The circadian oscillator has a pervasive influence upon plant cells. Approximately one-third of *Arabidopsis* transcripts are circadian-regulated (Covington et al., 2008). A variety of mechanisms communicate an estimate of time from the circadian oscillator to circadian-regulated cellular processes. For example, circadian rhythms of transcription likely arise from interaction of oscillator components or downstream factors with circadian-regulated and phase-specific *cis* elements across the genome (Harmer et al., 2000; Harmer and Kay, 2005; Covington et al., 2008). There may also be genome-wide circadian regulation of mRNA splicing (Sanchez et al., 2010). Post-translational processes including phosphorylation and poly ADP ribosylation, and also redox, Ca^2+^ and metabolite signaling participate in circadian regulation in *Arabidopsis* (Sugano et al., 1999; Panda et al., 2002; Dodd et al., 2007; Portolés and Más, 2010; Edgar et al., 2012; Haydon et al., 2013).

## Sugar Signals Regulate the Plant Circadian Oscillator

In the laboratory it is common to study circadian rhythms using constant conditions, following a duration of entrainment to 24 h cycles of light and/or temperature. Under constant conditions, the resultant oscillations reflect the dynamics and delays arising from interactions between the components of the circadian network. However, this does not reflect the environment of the Earth and most organisms experience repetitive daily light and temperature fluctuations due to the rotation of the planet. Daily rhythmic behaviors occurring in light/dark (LD) and temperature cycles often have different waveforms compared with constant conditions because the circadian clock responds to environmental signals. Light and temperature signals can also regulate rhythmic processes directly, in combination with circadian regulation (Dalchau et al., 2010).

**FIGURE 1:**
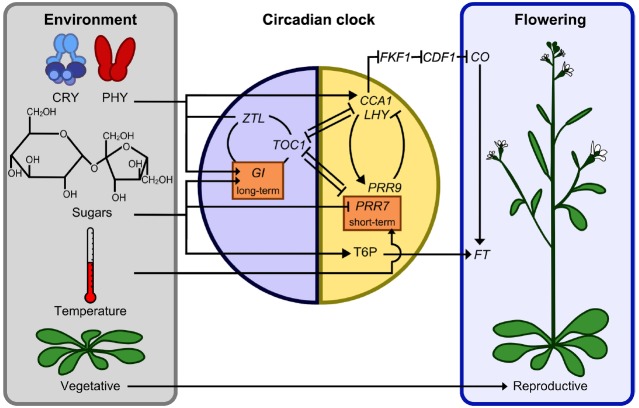
**Interactions between sugar and light signals, the circadian oscillator, and the photoperiodic regulation of flowering.** Entrainment cues comprising light signals derived from the cryptochrome (CRY) and phytochrome (PHY) photoreceptors, sugars produced by photosynthesis, and daily temperature fluctuations act upon the circadian clock through several intersecting pathways. The photoperiodic transition to flowering could be influenced by the action of sugars upon *PRR7* and *CCA1* transcription, and hence *CONSTANS* (*CO*), and also involve FT regulation by trehalose 6- phosphate (T6P) sugar signaling (Wahl et al., 2013), although the photoperiodic regulation of FT may not be entirely T6P-dependent. A reduced set of oscillator components are shown to emphasize those associated with circadian sugar signals. Morning/day-expressed components and evening/night-expressed components superimposed on the yellow and blue panels, respectively. Gene name abbreviations not described elsewhere: *ZEITLUPE* (*ZTL*), *TIMING OF CAB2 EXPRESSION1* (*TOC1*), *LATE ELONGATED HYPOCOTYL* (*LHY*), *FLAVIN-BINDING*, *KELCH REPEAT*, *F BOX 1* (*FKF1*), *CYCLING DOF FACTOR1* (*CDF1*).

Around dawn, the phase of the circadian oscillator is adjusted in response to low intensity light detected by photoreceptors as the sun rises. Later, as the light intensity increases, this is followed by a second entrainment event, in which the phase of the oscillator is advanced in response to the rhythmic accumulation of sugars that occurs every morning as a result of the daily activation of photosynthesis (Haydon et al., 2013). This so-called metabolic dawn occurs due to the sensitivity of morning-active components of the circadian oscillator to low concentrations of endogenous sugars. *PSEUDO-RESPONSE REGULATOR7* (*PRR7*) is repressed by sugars, whereas *CIRCADIAN CLOCK ASSOCIATED1* (*CCA1*) promoter activity is increased by sugars (Haydon et al., 2013; Figure 1). Inhibition of photosynthesis by maintaining plants in CO_2_-free air or by treatment with the photosynthetic electron transport inhibitor 3-(3,4-dichlorophenyl)-1,1-dimethylurea (DCMU) has the reverse effect, increasing *PRR7* expression and suppressing *CCA1* (Haydon et al., 2013). Since *PRR7* and *CCA1* are in a feedback loop active in the morning, this pathway appears to be responsible for entrainment to metabolic dawn, which is confirmed by the inability of *prr7-11* loss-of-function mutants to entrain to sugar signals (Haydon et al., 2013).

We suggest that metabolic dawn might co-ordinate the circadian regulation of metabolism with photosynthetic activity. The circadian oscillator can be entrained to very low light (<10 μmol m^–2^s^–1^; Covington et al., 2001). This suggests that the oscillator is set to the morning phase long before there is sufficient light to elicit significant photosynthesis, particularly in long summer days at high latitudes. Additionally, in very overcast days, significant photosynthetic activity might occur later in the day than in sunny days. Therefore, the temporal separation of dawn and peak photosynthetic activity depends upon the season and the weather. Through entrainment to sugars at a metabolic dawn the circadian oscillator might adapt the temporal regulation of metabolic outputs to accommodate environmentally-induced variability in the timing of photosynthesis, which contrasts with the timing of actual dawn that can be anticipated accurately. Thus, in sunny days an increase in sugars early in the photoperiod would advance the oscillator, potentially to a phase appropriate for driving processes associated with sugar metabolism. However, if sugars do not rise early in the morning, due to low photosynthesis, or rise only later in the day due to fluctuations in light availability, the oscillator will not be phase advanced because the oscillator loses its sensitivity to sugars toward the end of the photoperiod (Haydon et al., 2013).

Sugars also affect the circadian oscillator in a long term-response pathway that enhances circadian rhythms in Arabidopsis leaves in the dark and requires *GIGANTEA* (*GI*; Dalchau et al., 2011; Figure 1). This long-term response to sugars in the dark is unrelated to entrainment and its purpose is unknown. Sugar-mediated regulation of the circadian oscillator might occur to optimize the performance of the oscillator to control circadian timing, but also could be a mechanism by which sugars affect the timing of biochemical process that are considered outputs of the clock, because sugars can regulate circadian outputs directly without acting upon the core oscillator. Next, we consider ways in which sugars may interact with such circadian-regulated processes.

## The Role of Sugar Signals in Regulating Circadian Oscillator Outputs 1: Starch Metabolism

Not all carbon that is fixed during the day can be utilized immediately for growth. Instead, carbon must be stored to supply respiration and growth during the night. In plants such as Arabidopsis, the nocturnal demand for carbon is met by the consumption of starch that has been stored through the day. This cycle of storage and consumption involves an element of circadian control because in *cca1-11 lhy-21* double mutants the consumption of starch at night is faster than in wild types, meaning that all starch is consumed long before dawn (Graf et al., 2010). A multiscale model of plant carbon assimilation and growth has demonstrated that the correct accumulation of starch during the day is essential for optimal biomass accumulation, and compound effects mean that even a small starch under-accumulation can result in plants half the expected size (Chew et al., 2014).

The diel dynamics of starch production and consumption are linear. Starch accumulates almost linearly during the day, and similarly is consumed such that starch reserves are depleted in a linear manner. The rate of starch production and depletion is entrained by the photo- and skoto-period to ensure that starch quantity peaks at dusk, and the entire starch reserve is consumed at dawn (Graf et al., 2010). Hypotheses explaining the linearity of starch turnover and the anticipation of dawn and dusk have been generated through the formulation of mathematical models of starch dynamics in Arabidopsis. These models assumed that there is circadian regulation of starch metabolism, though currently the mechanisms are not clear. In one class of model, the circadian clock provides an estimate of the time until dawn that is incorporated into a biological arithmetical calculation to ensure the rate of starch degradation is inversely proportional to the time until dawn and is proportional to the remaining starch pool (Scialdone et al., 2013; Seaton et al., 2014). In another class of model, the rate of starch degradation is considered to be a function of circadian regulation with feedback from carbon availability adjusting the circadian phase of the rate of starch degradation such that the linear dynamics of starch production/consumption arise as an emergent property of the system (Feugier and Satake, 2013). The prediction that circadian phase can be adjusted by carbon availability (Feugier and Satake, 2013) has been confirmed experimentally (Haydon et al., 2013), but it remains to be determined whether oscillator entrainment by sucrose participates in starch homeostasis.

## The Role of Sugar Signals in Regulating Circadian Oscillator Outputs 2: Flowering

Sugars signals have been implicated in the photoperiod-dependent transition from vegetative to reproductive growth. Trehalose-6-phosphate (T6P) is synthesized from glucose-6-phosphate and acts as a cellular measure of sugar availability (Wahl et al., 2013). It was shown recently that T6P is required for the expression of the floral inducer protein FLOWERING LOCUS T (FT; Wahl et al., 2013; Figure 1). In Arabidopsis, FT is produced in the leaves in response to flowering-inducing long days. FT is subsequently translocated to the shoot apical meristem where it induces floral development genes (Corbesier et al., 2007). In addition to regulating the systemic induction of the flowering time signal from the leaves, T6P acts locally in the shoot apical meristem to regulate flowering time and floral patterning genes (Wahl et al., 2013). It is proposed that local action of T6P ensures that photoperiodic induction of FT in long days is co-ordinated with sufficient carbohydrate availability to provide resources for new organ production (Wahl et al., 2013). FT production is also dependent on the photoperiod through internal coincidence detection, which results in the production of a floral regulator dependent on the relationship of the internal phase of the circadian oscillator with the external phase of the light and dark cycle (Nagel and Kay, 2012). Since sugars affect the phase of the circadian oscillator and regulates the dynamics of the oscillator in the dark through *GI*, which is also a regulator of flowering time (Dalchau et al., 2011), it is possible that sugar-mediated regulation of circadian oscillator function could have consequences for photoperiodic detection. However, this has not been tested. If sugar-mediated adjustment of the circadian oscillator does impinge upon photoperiodic detection and contributes to the control of flowering, this might be acting as a measure of available and/or transportable carbon, because the circadian regulation of photoperiodic detection appears to occur in the vascular tissue (Endo et al., 2014).

## Circadian Signaling between Organelles

**FIGURE 2:**
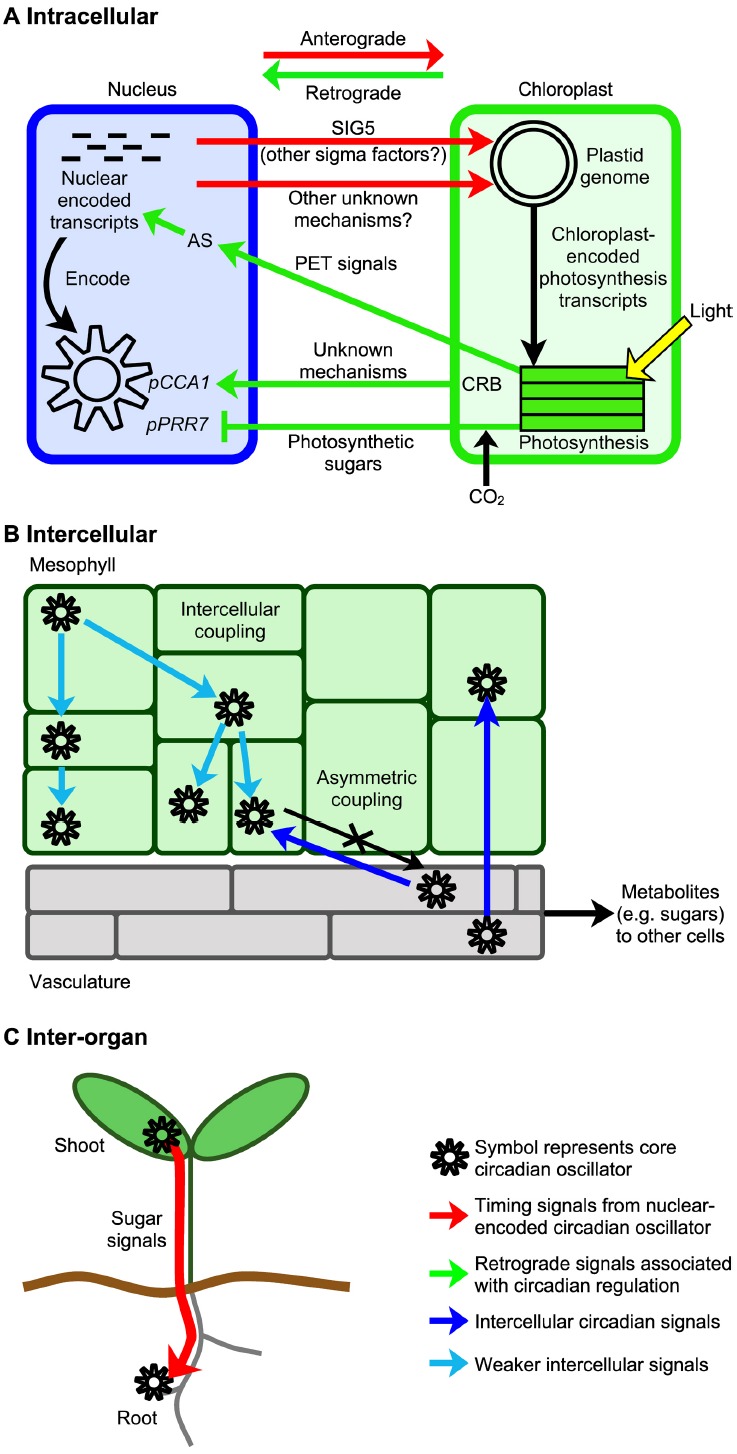
**Spatiotemporal circadian signaling in plants occurs across several scales and may involve metabolite signaling. (A)** Signal transduction processes communicate circadian timing information between the nucleus and chloroplasts, using mechanisms including sigma factors and metabolite signaling. There are circadian signals from the nucleus to chloroplasts, and from chloroplasts to the nucleus, which are termed anterograde and retrograde signals respectively. CRB is nuclear-encoded and proposed to act within chloroplasts (Hassidim et al., 2007). **(B)** Spatiotemporal waves of *CCA1* promoter activity move across leaves and provide evidence for weak intercellular coupling of the oscillators of neighboring cells (Wenden et al., 2012). The circadian oscillator of the vasculature influences the mesophyll circadian oscillator strongly, but not vice versa, suggesting asymmetric intercellular coupling (Endo et al., 2014). **(C)** A photosynthetic sugar signal may couple circadian oscillators between organs. Here, a sugar signal derived from the leaves is proposed to adjust the functioning of the circadian oscillator in roots (James et al., 2008). AS, alternative splicing, PET, photosynthetic electron transport-derived signals (Petrillo et al., 2014), and *pCCA1* and *pPRR7* indicate promoters of *CCA1* and *PRR7*, respectively.

In higher plants, there are circadian rhythms of photosynthesis that are controlled by the nuclear-encoded circadian oscillator (Hennessey and Field, 1991; Dodd et al., 2005). This implies that the nuclear-encoded circadian oscillator controls events within plastids through nucleus to plastid anterograde signaling (Figure 2A). Whilst anterograde circadian signaling appears to involve transcriptional regulators, retrograde circadian signaling pathways from plastids to the nucleus might involve metabolic signals such as those produced by photosynthesis (Figure 2A). Here, we focus upon the role of signaling in coupling of the circadian oscillator with chloroplasts. However, there are also oscillations in the abundance of nuclear-encoded transcripts for chloroplast-localized photosynthesis proteins (Harmer et al., 2000) that may contribute to the circadian regulation of chloroplasts.

A circadian signal is communicated to chloroplasts by a nuclear-encoded regulator of chloroplast transcription, SIGMA FACTOR5 (SIG5; Noordally et al., 2013; Figure 2A). *SIG5* is one of a family of 6 nuclear-encoded bacterial σ^70^-like subunits of plastid-encoded plastid RNA polymerase (PEP). In plants, sigma factors are regulators of chloroplast transcription that confer promoter specificity to PEP and are required for initiation of transcription. Each sigma factor is thought to regulate overlapping subsets of chloroplast genes (Kanamaru and Tanaka, 2004), forming an anterograde signaling network that provides nuclear control of chloroplast transcription. In Arabidopsis, circadian rhythms of nuclear-encoded *SIG5* drive rhythms of transcription from the blue light-responsive promoter of chloroplast-encoded *psbD* (Noordally et al., 2013) and potentially other chloroplast genes, communicating timing information to the chloroplast genome (Noordally et al., 2013). SIG5 alters the circadian rhythm of delayed chlorophyll fluorescence and increases photosynthetic efficiency in high light, so circadian regulation of this pathway may adapt photosynthesis to daily and transient fluctuations in the light environment (Nagashima et al., 2004; Noordally et al., 2013). Since around 70% of chloroplast transcripts can be circadian regulated (Noordally et al., 2013), circadian signaling by SIG5 likely forms one of several mechanisms connecting the circadian oscillator with chloroplasts.

Several retrograde signals may adjust the circadian oscillator in response to chloroplast metabolism. As discussed previously, sugars produced by photosynthesis adjust the transcription of circadian oscillator genes (Haydon et al., 2013). Since chloroplasts export carbohydrates in response to light-induced photosynthesis, sucrose-mediated oscillator entrainment forms a retrograde signal that communicates information concerning photosynthesis and chloroplast function to the circadian oscillator (Figure 2A).

Additional retrograde signals may also impact oscillator function (Figure 2A). First, photosynthetic electron transport generates a retrograde signal that adjusts the alternative splicing of nuclear-encoded transcripts encoding an SR protein (a regulator of RNA splicing) and other splicing factors (Petrillo et al., 2014). The signal is mobile and reaches the roots (Petrillo et al., 2014). The nature of the signal and whether it is circadian-regulated is unknown, but an interesting hypothesis is that circadian regulation of photosynthesis within chloroplasts may cause circadian modulation of a retrograde signal that alters nuclear mRNA splicing. Second, a chloroplast protein proposed to associate with plastid ribosomes (CHLOROPLAST RNA BINDING, CRB) alters the circadian amplitude of *CCA1* transcripts (Hassidim et al., 2007), which might involve retrograde signals including sugars (Atkins and Dodd, 2014).

## Circadian Signaling between Cells, Tissues, and Organs

In animals, the circadian system has a central pacemaker in the suprachiasmatic nucleus of the brain that communicates circadian timing information to peripheral oscillators within organs, using neuronal and hormonal signals (Mohawk et al., 2012). In plants, until recently the prevailing view was that circadian oscillators are locally-autonomous with a degree of cell type specialization (Thain et al., 2000; Wood et al., 2001; Dodd et al., 2004). However, recent advances demonstrate that interactions occur between the circadian clocks of cells of individual tissues, different tissues, and different organs. When seedlings are cultivated entirely under constant light (LL) and daily changes in *CCA1* promoter activity are measured using luciferase imaging, the leaves appear to be arrhythmic when the luciferase signal from the entire leaf is combined. However, high resolution imaging of the leaves of seedlings cultivated under LL reveals that spatiotemporal waves of *CCA1* promoter activity move across the leaves (Wenden et al., 2012). These are hidden when *CCA1::LUCIFERASE* activity is combined for the entire leaf, because different parts of the leaf are at different stages in wave progression, producing an overall measure that appears to be arrhythmic. The existence of the waves suggests there is local intercellular coupling of oscillator gene expression (Figure 2B; Wenden et al., 2012). There may also be intercellular coupling of root circadian oscillators (Fukuda et al., 2012). Intercellular circadian coupling is also indicated by the apparent dominance of the circadian oscillators of the vasculature over those in the mesophyll (Figure 2B; Endo et al., 2014). The nature of the signals that couple the oscillators between cells within leaves are unknown, but the short or long term effects of sugar upon the circadian oscillator form strong candidates (Dalchau et al., 2011; Haydon et al., 2013). Alternatively, intercellular oscillator coupling in plants could involve signaling ions, mobile proteins or RNAs, and operate through apoplastic or symplastic pathways.

Plant circadian oscillators may be coupled between organs. Circadian rhythms occur in roots that are isolated from light. In LD, shoot and root circadian oscillations are synchronized. However, under LL the root circadian rhythm becomes desynchronized from the shoot circadian rhythm (James et al., 2008). Since a subset of oscillator transcripts in roots become disrupted by application of DCMU to leaves, a photosynthesis-associated signal may sustain root circadian function (Figure 2C). Moreover, under LD some oscillator transcripts in roots have greater sensitivity to sucrose than in leaves, suggesting sugar signals contribute to circadian oscillator function within roots (James et al., 2008).

## Conclusion

Recent breakthroughs have demonstrated the integration of plant circadian clocks with metabolism. It seems that the interlocking of circadian regulation with carbohydrate biochemistry in terms of photosynthesis, starch accumulation and feedback to the circadian clock maximizes the efficiency of the capture and utilization of solar energy by plants. We propose that this optimizes plant performance in an environment that is characterized by predictable changes, such as those arising from the days and seasons, and unpredictable changes, such as clouds passing across the sun. Since the biomass accumulated by photosynthesis and carbohydrate metabolism forms the basis of our food supply, existing and future studies of the interconnectivity of circadian clocks and metabolism across both daily and seasonal time, and subcellular and intercellular scales, are likely to make important contributions to future precision improvements in crops.

### Conflict of Interest Statement

The authors declare that the research was conducted in the absence of any commercial or financial relationships that could be construed as a potential conflict of interest.
